# Self-management knowledge, attitudes and practices among persons with type 2 diabetes in Ghana

**DOI:** 10.4102/phcfm.v17i1.4696

**Published:** 2025-03-14

**Authors:** Beatrice B. Johnson, Mary A. Jarvis, Jennifer A. Chipps

**Affiliations:** 1 Department of Nursing, Faculty of Community and Health Science, University of the Western Cape, Belville, South Africa; 2 Department of Nursing, School of Nursing and Midwifery, University of Health and Allied Sciences, Ho, Ghana; 3 Department of Nursing, Faculty of Nursing and Public Health, University of KwaZulu-Natal, Durban, South Africa

**Keywords:** diabetes type 2, self-care management, knowledge, attitudes and practices, information-motivation-behaviour skills model, Ghana

## Abstract

**Background:**

Diabetes is one of the major non-communicable diseases. Diabetes self-management has been identified as a key strategy to reduce complications and to improve health outcomes.

**Aim:**

This study aimed to investigate the diabetes self-management knowledge, attitude and practices among people with type-2 diabetes in Ghana.

**Setting:**

Two clinics for diabetes patients in the Ho municipality of Ghana were selected to conduct the study.

**Methods:**

An outpatient cross-sectional survey was conducted using a 57-item researcher-administered questionnaire based on the Information, Motivation, Behaviours Model adopted for Diabetes. A total of 321 patients with type 2 diabetes were randomly selected from the two outpatient clinics for diabetes in Ho, Ghana. Data were analysed using descriptive statistics and multiple linear regression modules were conducted to determine the predictors of self-management practices. Significance was set at *p* < 0.05.

**Results:**

The average score for knowledge was 11.37/24 ± 3.40 or 47%, indicating poor levels of diabetes self-management knowledge. Moderately positive attitudes were found (2.83/5 ± 1.57) [95% CI –1.86 to –3.80] with poor self-management practices with a median of 3.00 per week (maximum 5.20, minimum 0.60 per week). Knowledge explained 20% of variation in self-management practice.

**Conclusion:**

The findings from this study show an overall deficit in knowledge of diabetes with related low self-management practice. This suggests the need for robust self-management education programmes to improve access to diabetes self-management-related information.

**Contribution:**

This study highlights the important knowledge of diabetes in self-management.

## Introduction

The burden of diabetes and related complications impact on the individual, their family and the community.^1^ There is a global drive to reduce the complications associated with non-communicable diseases (NCDs) and diabetes in line with the Sustainable Development Goal (SDG) 3.4.^2^ Diabetes can be successfully managed to prevent complications^1^ such as cardiovascular and kidney problems,^3,4^ with self-management as the gateway to glycaemic control and improved quality of life.^5,6,7^ Self-management is the ability of individuals, families and communities to promote health, prevent diseases, maintain health and cope with illness and disability with or without the support of a healthcare provider.^8^ Self-management empowers and supports patients to commit to and sustain healthy behaviour by taking full responsibility for their care,^5,9,10^ and has been reported to reduce blood glucose levels. However, reports of adherence to diabetes self-management have been suboptimal,^11^ with non-compliance found to be high, especially in low- and lower-middle-income countries (LMICs).^3,12^

Self-management of diabetes is strongly influenced by the level of knowledge of diabetes self-management, attitudes towards diabetes self-management and self-management practice.^12,13^ In LMICs, low levels of self-management practices have been attributed to poor health literacy, inability to afford a healthy diet and a lack of support to maintain behaviour change.^14^ In Ghana, studies have reported a number of barriers to diabetes self-management. These include misconceptions linked to the cause of diabetes and the use of herbal medicines,^15^ over-restrictive dietary recommendations from health care professionals, lack of self-control over food choices, inadequate family support and side effects experienced from the medication.^15,16^

In this context, it is important to explore ways to ensure adherence to diabetes self-management programmes. Knowledge and the willingness to change, adopt and sustain healthy behaviour is crucial.^17,18^ Promoting change in behaviour can be enhanced through the use of behaviour theories that link knowledge, motivation and skills to actual behaviour, such as the Information-Motivation Behaviour (IMB) model.^17,18,19^ The IMB model is a social psychological theoretical model of healthy behaviour change developed by Fisher & Fisher, for human immunodeficiency virus (HIV)-related preventive behaviours.^20,21^ Over the years, the IMB model has been adopted for chronic disease management such as diabetes and hypertension.^18,21,22,23^ The IMB model posits that its constructs, information, motivation and behaviour were found to individually influence behaviour change in less complex behaviour.^24^ The interplay of these three constructs was found to enhance sustained, healthy behaviour change in people with type 2 diabetes,^25^ by translating accurate information into healthy behaviours, initiated and sustained through personal and social motivation.^18^ However, few studies have reported the contextual use of such theories in diabetes self-management in a LMIC such as Ghana. This study adopted the IMB ‘Skills model to explore the relationship between patients’ knowledge of diabetes, attitude and current practices of diabetes self-management practices.

According to the information construct of the IMB model, information related to a particular behaviour is needed to influence the decision to perform that healthy behaviour.^17,21^ In this study, information was termed ‘knowledge’. The level of knowledge of diabetes and self-management was assessed in the various domains of diabetes care with regards to the motivation construct of the IMB model; motivation is needed to influence the willingness of the person to enact healthy behaviour.^21^ Two dimensions of motivation, personal and social motivation need to interact to compel the individual to perform self-management activities. Motivation was termed ‘attitude’. Questionnaires on attitude towards diabetes self-management were used to assess the level of motivation to perform self-management activities. The behaviour skills construct of the IMB emphasises the importance for an individual to be equipped with the necessary skills to carry out self-management activities with ease. In this study, behaviour skills were termed ‘diabetes self-management activities’. The frequency of diabetes self-management activities performed in a week was assessed to determine the level of self-management activities. Therefore, the aim of this study was to investigate the diabetes self-management knowledge, attitude and practices among people with type-2 diabetes in Ghana comparing male and female differences.

## Research methods and design

An outpatient cross-sectional study (IMB) was conducted between May and August 2018 in Ghana. Using the IMB model, the study aimed to describe the levels of diabetes knowledge, attitude and self-management practices among patients with type two diabetes and the association or relationship between them.

### Setting

As most primary health care (PHC) in Ghana is provided through outpatient clinics, the research was carried out over a period of 4 months at the two outpatient clinics for patients with diabetes in the Ho Municipal Hospital and the Ho Teaching Hospital. The Ho municipality is bordered on the east with the Republic of Togo and is composed of a mix of sociocultural and multilingual groups. Ho Municipal Hospital offers primary care and a clinic for diabetes patients from Monday to Friday with an average monthly attendance of 900. Ho Teaching Hospital has an outpatients’ clinic for patients with diabetes which runs weekly on Thursdays with an average monthly attendance of 200. Clinics are managed by physicians and staffed by dieticians and registered nurses. Routine activities include monitoring of blood pressure, weight, blood glucose levels, lipid profiles and health education on self-management.

### Population and sampling

The study population consisted of adults living with type 2 diabetes who sought primary care at clinics for diabetes patients at the two selected outpatient clinics. The sample size of 384 patients was calculated for an infinite population using the formula below in equation 1:


$$\text{Infinite sample size }(n)={\text{Z}}^{2}\times P(1[C-p])/{\text{C}}^{2}$$ [Eqn 1]

Where:

*Z* = 1.75 (the Z score for a 92% confidence interval [CI])

*p* = 50%

*C* = 0.05 (5% margin of error).

Using the total registered diabetes population of 950 in both hospitals, the final minimum acceptable sample (266 patients) and 10% allowance for non-response (27 patients) were calculated (293 patients) using the formula in equation 2:


$$\text{Finite sample size}=\frac{n}{1+\frac{n}{N}}$$ [Eqn 2]

Where:

*n* = 302

*N* = 200.

A total of 325 patients with type-2 diabetes were selected from the clinic register using a systematic random sampling method (every other patient on the blood glucose register) from 26 May 2018 to 06 August 2018. Respondents were included in the study if they were diagnosed with type 2 diabetes, attended the outpatient clinics for diabetes management within the past 3 months and had at least three visits. This is to ensure that patients who regularly attend the study clinics are captured and were willing to give informed consent or assent to participate in the study. Those who were excluded from the study were patients who reported that they were sick at the time of the study and were incapable of the interview. Out of the 325 consenting patients with diabetes, 321 completed the questionnaires, constituting a 98.70% response rate.

### Instruments

A standardised researcher-administered questionnaire was developed from the constructs of the IMB model which informed the structure of the questionnaire. In this study, information (knowledge) construct of the model was developed using Starr County Patient’s Diabetes Knowledge Questionnaire. Motivation (attitude) dimension of the module was formed using the Diabetes Attitudes Survey, while behaviour skills (self-care activities) were developed using the Summary of Diabetes Self-Care (SDSCA)

The first section questions collected sociodemographic and health-related behaviour data (13 items). The second section included the validated 24-item scale, the Starr County Patient’s Diabetes Knowledge Questionnaire. The questionnaire was translated into Èʋegbe, the local dialect of the study area for easy comprehension. The validity of the Èʋegbe version of the 24-item scale for this study recorded a Cronbach’s alpha of α = 0.89.^26^ The scale was also validated in Pakistan and Malaysia with a Cronbach’s alpha of α = 0.702 and 0.757, respectively; indicating a valid and reliable tool for measuring diabetes knowledge. The scale was rated using ‘yes’, ‘no’, or ‘don’t know’, and questions were organised around knowledge on diet, causes, medication, foot care, complications and general knowledge of diabetes. An overall knowledge score was calculated out of 24 and classified as very good (above 75%), good (75% – 50%) and poor (below 50).^27^

The third section of the questionnaire includes the validated 10-item scale, the Diabetes Attitudes Survey. The internal consistency value for this study was Cronbach’s alpha of α = 0.68.^28^ The scale has a 5-point Likert scale (1 = Strongly Disagree; 5 = Strongly Agree) rating self-assessed attitudes with a total score of 50. The scale was combined to generate a composite score for measuring the attitude of patients with diabetes, ≤ 20% was considered ‘highly insufficient’, 21% – 40% ‘insufficient’, 41% – 60% ‘sufficient’, 61% – 80% ‘satisfactory’ and > 80% ‘highly satisfactory’.^28^ Two subscales were reported, with eight items measuring attitudes towards self-management and two attitudes towards the need for others to support them to care for their diabetes.

The last section of the questionnaire included the 10-item self-management practices scale (SDSCA) which had a moderate Cronbach’s alpha of α = 0.56 in this study,^29^ with 5 subscales measuring dietary management, exercise, medication adherence, glucose monitoring and foot care. The scale was scored on the number of days per week respondents performed self-management practice, scoring a total of seven for each item. In addition, the 11th item of SDSCA was ‘Have you smoked a cigarette – even one puff – during the past seven days?’ However, during the review of the instrument used for data collection, experts in diabetes care identified that alcohol consumption was peculiar to the research setting; hence, the suggestion was made to assess alcohol consumption instead of smoking because alcohol consumption was a risk factor for NCD over smoking in the research setting.^30^ Therefore, ‘How many “quarter piece” bottles of alcohol did you take during the past seven days?’ was adapted to the study setting and reported independently of the data under the sociodemographic characteristics.^11^ Subgroups of self-management practice were analysed and reported to reflect standard practice.^31^

### Data collection procedures

Administrative approval was obtained from the Volta Regional Director of Health Services, the medical directors of the Ho Teaching Hospital and the Ho Municipal Hospital, prior to the commencement of the study. Based on the calculated sample size, 325 patients with diabetes were randomly selected from 26 May 2018, until the estimated numbers were achieved on 06 August 2018. The patients’ blood glucose register was used to systematically select every second patient who agreed to participate in the study based on the inclusion criteria. The content of the information was explained in simple terms to the respondents in English and Èʋegbe, after which they were guided to sign the consent form. Those who could not read and write were assisted to thumb print the consent form. Depending on the respondents’ preferences, the questionnaire was researcher-administered in English or Èʋegbe in a private consulting room or patient treatment room to ensure privacy. Most respondents preferred to answer the questionnaire at the back of the waiting area for privacy. The questionnaire was completed within 30–35 min while respondents were waiting for their turn to check their vital signs or when waiting for their health education and medical consultation.

### Statistical analysis

Data were cleaned and analysed using an IBM Statistical Package for the Social Sciences (SPSS) software package version 25. Chi-square test statistics was used to examine the associations and differences in the study variables such as demographic, clinical data and knowledge levels by comparing the observed and expected counts between males and females with the *p*-value set at < 0.05 for significance. Descriptive statistics were calculated to describe categorical variables, whereas means and standard deviations were used for continuous variables. Multiple linear regression was performed to assess the impact of knowledge, attitudes and selected variables on days of practice of self-management.

### Ethical considerations

Ethics approval was received from the University of Western Cape Research Ethics Committee (No. BM17/10/2) and the University of Health and Allied Sciences Research Ethics Committee (No. UHAS-REC A.6 [13] 17 -18). Permission was received from the selected health facilities. We informed respondents of their right to opt out of the study at any time they chose without fear of reprisal. Further, questionnaires were administered at times convenient to the respondents. Anonymity was maintained by assigning codes to each participant to ensure confidentiality. No compensation for participation was provided.

## Results

### Demographic and clinical health data

Table 1 reports the demographic and clinical data of respondents. Out of the 325 consenting patients, 321 completed the questionnaires (98.70% response rate). About two-thirds of the respondents (214, 66.70%) were females, with age ranges from 30 years to 89 years (mean age of 57 ± 9.59 years). Two-thirds (203, 63.24%) resided in urban areas. More females than males lived alone (80, 37.38%, and 11, 10.2%, *χ*^2^ = 25.80, *p* = 0.001) and 230 (71.70%) lived with their partners. The majority of the respondents had received formal education (260, 81.0%), with nearly two-thirds of them attaining tertiary education (95, 29.60%). Less than half of the respondents were employed (155, 48.30%) with significantly more males than female respondents being employed males (58, 54.20%) vs. females (98, 45.32%), *χ*^2^ = 31.26, *p* = 0.001). Less than a quarter (58, 18.50%) of respondents reported that they consumed alcohol, with a significant higher proportion of male respondents than females reporting this (34, 31.77% vs 24, 11.21%: *χ*^2^ = 20.37, *p* = 0.001) (Table 1).

**TABLE 1:** Sociodemographic, review and clinical characteristics of respondents by gender.

Variables	All *n* = 321 (100%)				Female *n* = 214 (66.67%)				Male *n* = 107 (33.33%)				Test	*P*
	*n*	%	Mean	s.d.	*n*	%	Mean	s.d.	*n*	%	Mean	s.d.		
Urban residence	203	63.24	-	-	131	61.21	-	-	72	67.30	-	-	*χ*^2^ = 1.13	0.287
**Age m (±) years**	-	-	57.10	±9.59	-	-	56.53	±9.72	-	-	58.00	±9.28	*T* = 1.56	0.119
40–59 years	187	58.44	-	-	133	62.40	-	-	54	50.50	-	-	*χ*^2^ = 4.00	0.045*
Living with partner	230	71.70	-	-	134	62.60	-	-	96	89.71	-	-	*χ*^2^ = 25.80	0.001*
**Educational level**														
None	61	19.00	-	-	45	21.00	-	-	16	14.95	-	-	*χ*^2^ = 5.41	0.144
Basic	95	29.60	-	-	64	29.90	-	-	31	29.00	-	-	-	-
Secondary	70	21.81	-	-	50	23.40	-	-	20	18.70	-	-	-	-
Tertiary	95	29.60	-	-	55	25.70	-	-	40	37.40	-	-	-	-
**Employment**														
Employed	155	48.30	-	-	97	45.32	-	-	58	54.20	-	-	*χ*^2^ = 31.26	0.001*
Pension	48	15.00	-	-	19	8.90	-	-	29	27.10	-	-	-	-
Unemployed	118	36.80	-	-	98	45.80	-	-	20	18.70	-	-	-	-
**Review frequency**														
< Once a month	23	7.20	-	-	13	6.10	-	-	10	9.30	-	-	-	-
Once a month	285	88.80	-	-	189	88.31	-	-	96	89.70	-	-	-	-
> Once a month	13	4.00	-	-	2	5.60	-	-	1	0.93	-	-	*χ*^2^ = 4.93	0.085
Alcohol consumption	58	18.50	-	-	24	11.21	-	-	34	31.77	-	-	*χ*^2^ = 20.37	< 0.001*
Clinic review travel time (m, ±)	-	-	30.81	±30.65	-	-	33.46	±33.46	-	-	25.50	±23.25	*T* = 2.28	0.028*
**Clinical characteristics**														
Duration of diabetes (m, ±)	315	98.10	5.57	±4.40	208	97.10	5.57	±4.57	107	34.00	5.58	±4.05	*T* = 0.02	**0.985**
Received counselling	317	98.75	-	-	210	98.13	-	-	107	100.00	-	-	*χ*^2^ = 2.25	0.155

Note: *χ*^2^, Chi-square test; s.d., standard deviation;

* , Significance set at *p* < 0.05.

On average, respondents have lived with diabetes for 5.57 years (±4.40). The majority of the respondents (285, 88.80%) reported monthly to the clinic for their health review. Nearly all the respondents (317, 98.80%) reported they had been previously counselled on diabetes self-management by healthcare professionals (Table 1).

### Knowledge of diabetes

The respondent’s levels of knowledge on diabetes are reported in Table 2. The average knowledge score was 11.37/24 (±3.40) or 47.40%, with more than half of the respondents (171, 53.30/100) classified as having poor levels of knowledge. There was variation in knowledge levels by knowledge domains with the highest scores for Knowledge in dietary management (1.87/2 (±0.41), 93.50%), and most classified as having a good level of knowledge in dietary management (299, 93.20/100). Knowledge of foot care practice had the second highest knowledge score (1.40/2 (±0.54), 71.50%), with more than half of the respondents (168, 52.30%) classified within the range of good level of knowledge. Knowledge of diabetes complications was the third-highest scored knowledge domain with an average score of 4.91/9 (±1.65), 54.55%) (Table 2) and less than half of the respondents (145, 45.2%) had a good level of knowledge of diabetes complications. Knowledge of the causes of diabetes was scored the third lowest (2.08/4 (±1.17), 52.00%), with less than half of the respondents 133 (41.60%) had very good level of knowledge of causes of diabetes. Knowledge of diabetes medication was the second lowest domain recording (0.55/2 [± 0.69], 27.50/100), with over half of the respondents (182, 56.70%) having a poor level of knowledge and significantly more male respondents lacking knowledge of diabetes medication than female respondents (0.6/2 (±0.76), 34.50% vs 0.48/2 (±0.65), 24.00%), (*T* = -2.51, *p* = 0.009). Nearly all of the respondents (284, 88.50%) had poor general knowledge on diabetes (1.27/5 [±1.06], 25.40%).

**TABLE 2:** Diabetes knowledge of respondents by gender.

Knowledge scores	All (*n* = 321)				Female (*n* = 214)				Male (*n* = 107)				Test	*P*
	Mean	s.d.	*n*	%	Mean	s.d.	*n*	%	Mean	s.d.	*n*	%		
**Total average diabetes knowledge score/24 (±) (100%)**	11.37	±3.40	-	47.40	11.13	±3.22	-	46.40	11.87	±3.63	-	49.46	*T* = -1.87	0.063
Very good	-	-	26	8.10	-	-	13	6.10	-	-	13	12.10	*χ*^2^ = 5.42	0.066
Good	-	-	124	38.60	-	-	79	36.90	-	-	45	42.10	-	-
Poor	-	-	171	53.30	-	-	122	57.00	-	-	49	45.80	-	-
**Diet/2 (100)**	1.87	±0.41	-	93.50	1.87	±0.40	-	93.50	1.85	±0.40	-	92.50	*T* = -0.483	0.629
Very good	-	-	3	0.90	-	-	1	0.50	-	-	2	1.90	*χ*^2^ = 1.57	0.439
Good	-	-	299	93.20	-	-	201	93.90	-	-	98	91.60	-	-
Poor	-	-	19	5.90	-	-	12	5.60	-	-	7	6.50	-	-
**Foot care/2 (100)**	1.40	±0.54	-	71.50	1.42	±0.52	-	71.50	1.46	±0.5	7	73.00	*T* = -0.586	0.559
Very good	-	-	146	45.50	-	-	93	43.50	-	-	53	49.50	-	-
Good	-	-	168	52.30	-	-	118	55.10	-	-	50	46.70	*χ*^2^ = 3.32	0.189
Poor	-	-	7	2.20	-	-	3	1.40	-	-	4	3.70	-	-
**Complications/9 (100)**	4.91	±1.65	-	54.55	4.84	±1.68	-	53.78	5.04	±1.60	-	56.00	*T* = -1.00	0.317
Very good	-	-	59	18.40	-	-	39	18.20	-	-	20	18.70	-	-
Good	-	-	145	45.20	-	-	97	45.30	-	-	48	44.90	*χ*^2^ = 0.01	0.994
Poor	-	-	117	36.40	-	-	78	36.40	-	-	39	36.40	-	-
**Causes/4 (100)**	2.08	±1.17	-	52.00	2.00	±1.14	-	50.00	2.2	±1.22	-	55.75	*T* = -1.67	0.091
Very good	-	-	133	41.60	-	-	84	39.30	-	-	49	46.20	*χ*^2^ = 1.93	0.384
Good	-	-	91	28.40	-	-	61	28.50	-	-	30	28.30	-	-
Poor	-	-	96	30.00	-	-	69	32.20	-	-	27	25.50	-	-
**Medication/2 (100)**	0.55	±0.69	-	27.50	0.48	±0.65	-	24.00	0.6	±0.76	-	34.50	*T* = -2.51	0.009*
Very good	-	-	37	11.50	-	-	18	8.40	-	-	19	17.80	-	-
Good	-	-	102	31.80	-	-	66	30.80	-	-	36	33.60	-	-
Poor	-	-	182	56.70	-	-	130	60.70	-	-	52	48.60	*χ*^2^ = 7.44	0.024*
**General diabetes knowledge/5 (100)**	1.27	±1.06	-	25.40	1.22	±1.02	-	24.40	1.36	±1.14	-	27.20	*T* = -1.15	0.250
Very good	-	-	12	3.70	-	-	6	2.80	-	-	6	5.60	-	-
Good	-	-	25	7.80	-	-	13	6.10	-	-	12	11.20	-	-
Poor	-	-	284	88.50	-	-	195	91.10	-	-	89	83.20	*χ*^2^ = 4.43	0.109

Note: *χ*^2^, Chi-square test; s.d., standard deviation;

* , Significance set at *p* < 0.05.

### Attitudes towards diabetes care and social support

The attitude score for diabetes care was 30.27/50 or 60.54% positive attitude, which was rated as sufficient. Male and female respondents had similar attitude scores of 60.72% and 60.46%, respectively. However, the average score for attitude towards diabetes self-management was lower (22.12/40 [±6.77] or 55.30%) indicating insufficient attitudes towards self-management. The attitudes related to the need for others to support them to care for their diabetes were positive (8.14/10 [±1.94] or 81.40%) positive attitudes, with similar attitude between male and female respondents (8.15 [±1.93] or 81.5% vs 8.10 [±1.10] or 81.00%) Table 3 shows the results of respondents’ attitudes towards diabetes care and social support.

**TABLE 3:** Respondents attitudes towards diabetes care by gender.

Attitudes towards diabetes care	All m/5 ± s.d. *n* = 321		Female m/5 ± s.d. *n* = 214		Male m/5 ± s.d. *n* = 107		*T*-Test	*P*
	Mean	s.d.	Mean	s.d.	Mean	s.d.		
**Attitude towards diabetes/40**	22.13	±6.76	22.07	±7.13	22.22	±5.10	0.190	0.846
Health care professionals should help patients make informed choices about their care plans	4.19	±1.10	4.22	±1.01	4.12	±1.27	0.750	0.453
A controlled diet and regular exercise help in the maintenance of blood glucose	3.99	±0.96	4.03	±0.94	3.91	±1.01	1.110	0.269
Diabetes affects almost every part of a diabetic person’s life	3.67	±1.45	3.74	±1.42	3.51	±1.51	−1.080	0.283
People who take diabetic pills should be as concerned about their blood sugar as people who take insulin	3.18	±1.73	3.20	±1.72	3.139	±1.75	−0.340	0.733
Diabetic patient with normal blood glucose level can eat with restrictions	2.68	±2.22	2.49	±2.24	3.07	±2.14	−2.230	0.027*
It is not important to have controlled blood sugar because the complications of diabetes will happen anyway	1.62	±2.02	1.49	±2.06	1.88	±2.06	−1.640	0.102
People whose diabetes is treated by just a diet must worry about getting many long-term Complications	0.99	±1.79	0.90	±1.71	1.18	±1.95	−1.300	0.193
People who take oral medications to treat their diabetes have the same level of disease as those who take insulin	0.43	±1.25	0.47	±1.30	0.35	±1.15	−0.850	0.396
**Attitude towards support for diabetes care/10**	8.14	±1.95	8.15	±1.94	8.10	±1.97	0.220	0.829
Support from family and friends are important in dealing with diabetes	3.95	±1.52	3.93	±1.54	3.98	±1.5	−0.259	0.796
Diabetic patient is more responsible than the doctor and family in the care of diabetes	3.63	±1.62	3.54	±1.70	3.81	±1.43	−1.440	0.150
**Attitude/50**	30.27	±6.87	30.22	±7.26	30.36	±6.04	0.170	0.864

Note: *T-T*est, Independent Samples; s.d., standard deviation;

* , Significance set at *p* < 0.05.

### Self-management practice and adherence

The results of self-management practice and adherence levels among respondents are presented in Table 4.

**TABLE 4:** Respondent’s average scores for diabetes self-management practice by gender.

Daily self-management practices per 7 days	All *n* = 321 m(±sd)/7 %standard				Female *n* = 214 m(±sd)/7 %standard				Male *n* = 107 m(±sd)/7 %standard				CI/Test	*P*
	Mean	s.d.	*n*	%	Mean	s.d.	*n*	%	Mean	s.d.	*n*	%		
**Dietary practices**	5.35	±1.85	140	43.50	5.42	±1.83	99	46.30	5.22	±1.87	41	38.000	0.91	0.364
													1.83	0.176
Avoid eating high fat foods such as fatty meat or full-fat dairy products, fried yam, fried eggs	5.85	±1.67	166	51.71	5.97	±1.60	120	56.07	5.62	±1.79	46	42.990	*T* = 1.80	0.073
													*χ*^2^ = 4.89	0.027*
Following a recommended healthful eating plan	5.72	±1.61	149	46.42	5.74	±1.61	103	48.13	5.67	±1.60	46	42.990	*T* = 0.34	0.731
													*χ*^2^ = 0.76	0.384
Eat three or more servings of fruits and vegetables including spinach soup and stew	4.48	±2.27	104	32.40	4.54	±2.29	74	34.58	4.37	±2.22	30	28.040	*T* = 0.61	0.543
													*χ*^2^ = 1.39	0.238
**Medication adherence**														
Taking your hypoglycaemic medication as prescribed by your health care provider	4.83	±2.90	180	56.07	4.68	±2.96	117	54.67	5.12	±2.77	63	58.880	*T* = -1.28	0.201
													*χ*^2^ = 0.51	0.474
**Foot care**	3.76	±2.90	117	36.41	3.58	±2.88	58	33.60	4.14	±2.90	47	43.000	*T* = 1.64	0.102
													*χ*^2^ = 9.17	0.003
Inspect the inside of your shoes or slippers for stones, moist	3.93	±2.87	122	38.01	3.66	±2.89	73	34.11	4.46	±2.78	49	45.790	*T* = -2.35	0.019
													*χ*^2^ = 4.13	0.042*
checking your feet for discolouration, abrasions	3.62	±2.92	112	34.89	3.51	±2.88	69	32.24	3.82	±3.02	43	40.190	*T* = -0.89	0.374
													*χ*^2^ = 1.98	0.159
**Exercise (Std: ≥ 3 days)**	2.38	±2.12	140	45.47	2.34	±2.08	93	43.22	2.46	±2.15	47.85	87.850	*T* = 0.48	0.630
													*χ*^2^ = 0.01	0.937
Participate in at least 30 min of physical activity (continuous activity, including walking, weeding	3.66	±2.33	223	69.47	3.58	±2.24	147	68.69	3.82	±2.49	76	71.030	*T* = -0.88	0.379
													*χ*^2^ = 0.18	0.668
Participate in a specific exercise session (such as health walk, gym other than what you do around the house or as part of your work	1.10	±1.88	56	17.45	1.10	±1.92	38	17.76	1.10	±1.82	18	16.820	*T* = -0.02	0.983
													*χ*^2^ = 0.04	0.853
**Glucose Monitoring (Std > 1 day)**	0.85	±1.70	97	30.21	0.75	±1.55	62	28.74	1.03	±1.97	35	33.200	*T* = -1.39	0.165
													*χ*^2^ = 4.69	0.030*
Testing your blood sugar by yourself	0.91	±1.79	99	30.84	0.80	±1.64	62	28.97	1.11	±2.06	37	34.580	*T* = -1.46	0.146
													*χ*^2^ = 1.05	0.305
Testing his/her blood sugar the number of times recommended by your health care provider	0.79	±1.62	95	29.60	0.70	±1.47	61	28.50	0.95	±1.89	34	31.780	*T* = 1.32	0.189
													*χ*^2^ = 0.37	0.545
**Overall Practice mean/7**	3.00	0.60–5.20	135	42.00	2.93	0.60–6.00	86	40.19	3.20	0.60–6.00	49	45.790	*U* = -2.21	0.027*
													*χ*^2^ = 8.72	0.003*

Note: Cl/Test, confidence interval/test statistic; *χ*^2^, Chi-square test; s.d., standard deviation;

* , Significance set at *p* < 0.05.

The average median of self-management practice per week was 3.00 with a maximum of 5.20 per week and minimum of 0.60. More male than female respondents recorded higher number of days on which they carried out self-management practice (3.20/7) with a maximum of 6.0 and a minimum of 0.6 days per 7 days versus 2.93/7 days, maximum of 6.0 and a minimum of 0.6 days per 7 days (*U* = –2.21, *p* = 0.027).

The self-management practice days varied by different practices. Dietary management was the highest reported self-management practice (5.35 [±1.85] days out of the 7 days), with a similar pattern with male and female respondents (5.22 ± 1.87 vs 5.4 ± 1.83) days per week (*T* = 0.91, *p* = 0.364) (Table 4). Medication adherence was the second-highest reported self-management practice with an average of 4.83 ± 2.90 days a week. Over half of the respondents (180, 56.1%) adhered to their medication every day as prescribed with no significant differences between males and females (63, 58.9% vs 117, 54.7%). Foot care was reported as the third highest self-management practice at an average of 3.76 ± 2.90 days a week. Less than half of the respondents (117, 36.4%) adhered to foot care with significant differences between males and females (43% vs 33.60%, *p* = 0.003). The most practised foot care practice being ‘inspecting the inside of your shoes or slippers for stones and moisture’ (3.93 ± 2.87 days per week). More male respondents reported inspecting their feet than females (4.46 ± 2.78) days vs 3.66 ± 2.89 days, *p* = 0.019).

Exercise was the second-lowest self-management activity performed on an overall average of 2.38 ± 2.12) days per week. Nearly three-quarters of the respondents (223, 69.5%) adhered to at least 30 min of physical activity 3 days or more a week with the most performed exercise activity being ‘engagement in at least 30 min of physical activity (continuous activity, including walking)’ on an average of 3.66 ± 2.33 days a week. Glucose monitoring was the lowest-rated practice being performed less than 1 day a week (0.85 ± 1.70), significant differences existed between male and female respondents adherence to glucose monitoring (33.2% vs 2.8.74%, *p* = 0.030). Only 95 (29.6%) respondents adhering to daily recommended glucose monitoring by healthcare professionals.

### Predictors of diabetes self-management practice

Multiple linear regression was performed to assess the impact of knowledge, attitudes, gender, age, rurality, marital status, educational status, employment status and duration of diabetes on days of practice of self-management. The full model containing all predicators was statistically significant (*r*^2^ = 0.164, *F*(9.302) = 7.793, *p* < 0.001). The model as a whole explained 16.4% (adjusted R squared) of the variance in days of self-management practice, with knowledge of diabetes, educational and employment status contributed significantly to self-management practice. Knowledge of diabetes had a positive and significant impact on practice, explaining 20.8% of the variance in practice days (*B* = 0.208, *t* = 3.508, *p* = 0.001), followed by employment (*B* = –0.159, *t* = –2.533, *p* = 0.012) and educational status (*B* = 0.157, *t* = 2.389, *p* = 0.018) (Table 5).

**TABLE 5:** Predictors of diabetes self-management.

Variables	*B*	SEB	C/I		*t*	*P*
Self-management practice	-	-	-	-	4.265	0.000
Location	0.096	0.956	−0.204	−3.559	1.754	0.080
Gender	0.060	0.997	−0.892	−3.033	1.073	0.284
Age	0.040	0.964	−1.251	−2.542	0.670	0.504
Marital status	−0.079	1.090	−3.625	−0.664	−1.358	0.175
Educational status	0.157	0.505	0.213	2.200	2.389	0.018
Employment status	−0.159	0.580	−2.609	−0.328	−2.533	0.012
Duration of diabetes	0.036	0.513	−0.663	−1.357	0.676	0.500
Knowledge of diabetes	0.208	0.141	0.217	0.772	3.508	0.001
Attitude towards diabetes care	0.045	0.055	−0.071	−0.184	0.866	0.387

Note: *B*, standardised coefficient beta; SEB, Standard error for the unstandardized beta; C/l, confidence interval; *t*, test statistic; *P*, significance set at *p* < 0.001.

## Discussion

This study aimed to investigate knowledge, attitudes and practices among people with type 2 diabetes using the IMB model of Information (knowledge of diabetes), Motivation (attitude towards diabetes care) and Behaviour. The findings suggested that respondents had positive attitudes towards diabetes management but poor knowledge of diabetes and poor diabetes self-management practices. Also, a significant association existed among Knowledge, attitude and practice (KAP). The low level of knowledge reflected in the low levels of self-care practice even though overall attitude was reported to be positive, confirming the role of knowledge in self-care activities.

Knowledge of diabetes is one of the key elements in diabetes self-management with glycaemic control reported positive correlation with knowledge.^32,33,34^ This study found poor levels of knowledge with only 47.4% of respondents with adequate knowledge, confirming several studies that reported low knowledge levels in high- and low-resource settings.^35,36,37,38^ This finding is of concern because the majority of respondents had formal education and visited the clinic for diabetes once a month for routine care. Notwithstanding, another study among the respondents of the current study reported elsewhere,^39^ suggested low coverage of core domains of diabetes care and self-management during diabetes education during routine clinic visits. This was attributed to the lack of trained diabetes educators and the absence of guidelines on diabetes education. However, a study in North Ethiopia reported higher levels (70.4%) of diabetes self-management-related knowledge among persons living with diabetes.^40^ This difference may be attributed to different tools and settings or levels of literacy and increased education sessions at the clinic for diabetes patients in North Ethiopia. However, in this study, knowledge did vary by different domains. Dietary management (93.5%) and knowledge of foot care (71.5%) had high scores with lower scores for complications from diabetes (54.6%) and causes of diabetes (52%). Of concern was that medication adherence (27.5%) and general knowledge about diabetes (25.4%) had very low scores which again was confirmatory with other studies.^28,35^

According to the IMB model, a well-informed individual needs personal and social motivation to influence the enactment of recommended behaviour.^21^ The study suggests positive good levels of motivation,^28^ with positive attitudes towards diabetes self-management reported by 73.7% of the respondents. This study aligns with a study conducted in Ethiopia that reported 70.4% of respondents had a good attitude towards diabetes self-management.^40^ In contrast, another study conducted in a different setting showed that 70.0% of respondents had poor attitudes towards diabetes management.^41^ These differences in the findings may again be attributed to the difference in the research tool used for the data collection. In assessing the attitude of respondents towards self-management support, most of the respondents (81.40%) were optimistic that support from family and friends was important in dealing with diabetes, which was higher than a study conducted in Ethiopia (54.9%) for social support for diabetes self-management.^42^ This finding indicates that patients with diabetes perceived to receive support from their family and friends in managing diabetes. This is a positive indicator for effective self-management as studies from different setting reported a positive relation between social support and adherence to self-management practice and subsequent glycaemic control.^43,44^ Tailored family-centred diabetes educational programmes will go a long way to improve care and quality of life of people with diabetes.^45^

Self-management practice was measured by frequency of practices per week against recommended practice adherence per week.^46^ The frequency of self-management practices was low, with a reported average of 3.0 days practice a week which concurred with studies done in the United Arab Emirates and Egypt,^37^ but lower than the 5.06/7 days reported in Ethiopia.^47^ Dietary management was the most frequent self-management practice at 5.35/7 days ±1.85. This was higher than in similar studies conducted in Ghana (4.40/7 days ±1.52),^11,48^ in Zimbabwe (3.64 days ±1.82),^49^ and in Ethiopia (5.06 days a week).^47^ The most adhered-to dietary plan was that of a low-fat diet (5.85/7 days ±1.67) with 166 (51.7%) of respondents adhering to this practice. Eating three or more servings of fruits and vegetables, including spinach soup and stew, per week was rated the lowest (4.48/7 days ± 2.27) but was higher than similar studies.^11,37,49^ The high dietary adherence in this study may be attributed to the high proportion of patients (98.75%) who received counselling from healthcare professionals with the health education normally including information on diet.

Even though knowledge on medication was low, medication adherence was the second highest practice at 4.83 days a week ±2.90, and a reported 180 (56.1%) respondents adhered to taking medication daily. This is similar to other studies across Africa,^50,51^ again this is possible because medication adherence is a major focus in health education. Foot care was practised 3.76 days ± 2.90 a week which was lower than 5.26 days a week reported among people with type 2 diabetes in Ethiopia,^47^ and higher than in a similar study in Ghana (2.86 days a week ±2.16).^11^ Of concern is, exercise was done for an average of 2 days a week. This was similar to studies done in Pakistan.^11,37^ However, this finding contrasts with the 4.37 days of exercise recorded by Mogre et al. in Tamale, Ghana.^11^ Exercise adherence is challenging in LMIC with significant variations because of different patient characteristics, socioeconomic factor and lack of motivation among others.^52^

Though glucose monitoring best practice is recommended for every day, in this study this was done only about once a week. These findings were lower than in another setting in Ghana with reports of twice a week,^11^ similar to a study conducted by Moore et al.^11^ Males monitored their glucose more regularly than female respondents. Both findings might be because of financial reasons on account of low ownership of blood glucose monitoring machines and the cost of testing strips.^53,54^

Lastly, in considering factors that may influence self-management practices, high levels of knowledge and positive attitudes have been found to enhance diabetes self-management practices.^11,12,14,53,54^ This was also confirmed in this study where knowledge level significantly predicted self-management practices, confirming knowledge of diabetes has the potential to enhance diabetes self-management.^13,34,52,55^ Besides, this study found that, social demographics such as educational status and employment are likely to influence self-management practice of individuals with diabetes. This result is not limited to Ghana; a study in Ethiopia established that, educational status may influence good diabetes self-management practice.^53^ This findings suggests that people with higher educational status are more likely to seek for and understand diabetes-related information and are more likely to engage in diabetes self-management. Similarly, employment status has implication for affording healthy diet and medication. The findings from this study have implications for increase access to diabetes-related information by patients and support for self-management of persons with diabetes.

### Recommendations

The study contributed to understanding the role of knowledge on self-care management of chronic diseases such as diabetes and provides strong support for strengthening knowledge as a core component in self-care management interventions.

### Strengths and limitations

This study is the first study in Ghana to employ the IMB model to explore the knowledge, attitude and practices of persons living with diabetes self-care management. However, the study was only conducted on people with diabetes in a selected region and who attended hospital outpatient facilities. Although the study tool was translated into Èʋegbe, some items might not reflect the meaning of the original tool. It might also not have captured socio-cultural dimensions of knowledge, attitude and practice of diabetes self-management in the context of the study setting. However, this study provided vital information on the state of diabetes self-management in the Ho municipality of Ghana which can inform self-management educational programmes.

## Conclusion

The findings from this study show an overall deficit in knowledge of diabetes with related low self-management practice. These have implications for glycaemic control. Innovative diabetes self-management education programmes are needed to equip patients with type-2 diabetes with information on diabetes and its self-management.
